# Factors Associated With Self-Selected Step Rates Between Collegiate and High School Cross Country Runners

**DOI:** 10.3389/fspor.2020.628348

**Published:** 2021-01-26

**Authors:** Lace E. Luedke, Mitchell J. Rauh

**Affiliations:** ^1^Department of Kinesiology, University of Wisconsin Oshkosh, Oshkosh, WI, United States; ^2^Doctor of Physical Therapy Program, San Diego State University, San Diego, CA, United States

**Keywords:** running injury, cadence, stride frequency, epidemiology, athletics, running, distance running

## Abstract

**Introduction:** Cross country is a popular high school and collegiate sport with a high rate of running-related injuries (RRI). Among high school runners, higher step rates have been associated with greater running experience and decreased body height, and lower step rates have been prospectively associated with increased risk of shin RRI. These associations have not been reported in collegiate cross country runners. The purpose of this study was to compare step rates between collegiate and high school cross country runners. Secondary objectives included determining if step rates in collegiate runners were related to experience and anthropometric variables, and whether their self-selected step rates were prospectively related to lower extremity RRI.

**Materials and methods:** Twenty-nine NCAA Division III collegiate cross country runners (13 females, mean ± SD age 19.7 ± 1.3 years) completed a survey and ran at their self-selected speed. Step rate was assessed with Polar RCX5 wristwatches and S3+ Stride Sensors™ on the first day of the season. Runners were followed during the season for occurrence of time-loss lower extremity RRI. A cohort of 68 high school runners was used for comparison of step rates at their self-selected speeds.

**Results:** Collegiate runners' self-selected step rates (177.1 ± 7.2 spm [steps per minute]) were higher than high school runners' (171.3 ± 8.3 spm) (*p* = 0.01). Collegiate runners ran at higher self-selected speeds (4.6 ± 0.5 m/s) than the high school runners (3.8 ± 0.5 m/s) (*p* < 0.001). A lower percentage of collegiate runners ran at ≤166 spm than high school runners. Body mass was negatively correlated with step rate in collegiate runners. During the season, 41.3% of collegiate runners experienced lower extremity RRI. Step rates for collegiate runners who did not experience RRI (178.9 ± 7.7 spm) were not significantly higher than runners who did experience RRI (174.5 ± 5.7 spm) (*p* = 0.10).

**Discussion:** Higher step rates were found in collegiate than high school runners, but the difference was partially explained by higher self-selected running speeds. Thus, variations in step rate between high school and collegiate runners may be expected based on experience, speed, and body mass.

## Introduction

Cross country is a popular high school and collegiate sport. In 2018, 488,640 runners participated in high school cross country (National Federation of State High School Associations, 2019) and 29,945 runners participated in collegiate cross country through the NCAA® (National Collegiate Athletic Association, 2018–2019). However, cross country has a high rate of running-related injuries (RRI) to the lower extremity among participants (Rauh et al., 2006; Yang et al., 2012; Roos et al., 2015). In a study of over 400 high school cross country runners from 23 different schools, an overall time-loss RRI rate of 17.0/1,000 athletic exposures (AEs) was observed during the season (Rauh et al., 2006). Collegiate cross country and track & field are among the sports with the most overuse injuries with NCAA Division 1 athletes in these sports experiencing 8.3–17.6 overuse injuries/10,000 AEs (Yang et al., 2012). When collegiate cross country athletes were analyzed separately from track & field athletes, both men and women were among the highest rates of overuse injury among college athletes (13.7–19.6/10,000 AEs) (Roos et al., 2015). Approximately 50% of collegiate cross country runners reported a history of leg pain that caused them to miss cross country practice and affected race performance (Reinking et al., 2007).

Step rate is a measure of the number of steps, or total foot contacts, taken per minute. Alterations in running step rate have been associated with changes in lower extremity joint loading and braking forces (Heiderscheit et al., 2011). Step rates may be affected by years of training experience as 10 male collegiate track runners increased their step rates at set speeds over 2–4 years of participation (Nelson and Gregor, 1976). Trained runners with half-marathon personal best times of 70–86 min displayed higher step rates than untrained active participants when running at the same speeds from 2.78 to 4.72 m/s (Gomez-Molina et al., 2017) While running at 4.84 ± 0.36 m/s, highly trained runners of the French national marathon team ran at higher step rates than well-trained national runners or non-trained students who ran occasionally; the non-trained students also had higher body mass than the highly trained runners (Slawinski and Billat, 2004).

In prior studies in adult runners, step rate has been associated with anthropometric variables, including body mass and height. When speed was fixed, individuals with greater height displayed longer step lengths and lower step rates (Elliott and Blanksby, 1979). Cavanagh and Kram (1989) found that taller & heavier male runners generally had longer stride lengths, and thus lower step rates, at 3.83 m/s. However, correlations between individual anthropometric variables and stride parameters were not statistically significant. Similar patterns have been observed in elite triathletes running at speeds of roughly 5 m/s (Landers et al., 2011). Among high school cross country runners, higher step rates were significantly associated with greater running experience and less body height (Luedke et al., 2018).

Step rate manipulation via gait retraining has resulted in reduced loading rates in runners (Wang et al., 2020) and is an effective intervention in the treatment of RRIs like patellofemoral pain (Bonacci et al., 2018; Esculier et al., 2018). Because of the relationships between step rate and joint loading, self-selected step rates may influence the risk of RRI. To our knowledge, in the first prospective study to assess the relationship between step rate and RRI in high school cross-country runners, lower step rates were associated with increased risk of shin RRI that limited or stopped running participation for one or more days of practice or competitive events (Luedke et al., 2016). In a more recent prospective study, the step rate of adult runners 18–50 years old during a 2.0–3.1 mile run at their self-selected pace was not significantly linked with RRIs that limited activity for 7 or more days whether they required medical consultation or not (Szymanek et al., 2020). The reasons for differences in findings may be due to RRI definition or perhaps step rate may be a risk factor for some injury types or only in specific running populations.

Presently, associations between step rate and experience, height, and body mass have not been reported in collegiate cross country runners. Therefore, the purpose of this study was to assess these relationships and then compare step rates between collegiate and high school cross country runners. We hypothesized that collegiate runners would run at higher step rates than high school runners as collegiate runners were expected to have greater running experience and run more frequently at higher speeds. Secondary objectives included determining if step rates in collegiate runners were related to experience and anthropometric variables, and whether step rates differed between injured and non-injured runners during the season.

## Materials and Methods

### Participants

Twenty-nine NCAA Division III collegiate cross country runners (13 females and 16 males, mean ± SD age 19.7 ± 1.3 years) were prospectively followed during an intercollegiate season. Members of the university's women's and men's cross country teams without current RRI were invited to participate in the study. Participants were cleared for full participation and were free of any injuries limiting their participation in athletic activities at the time of assessment. The study protocol was approved by the university's Institutional Review Board. All participants provided informed consent prior to data collection.

### Study Protocol

**Figure 1:**
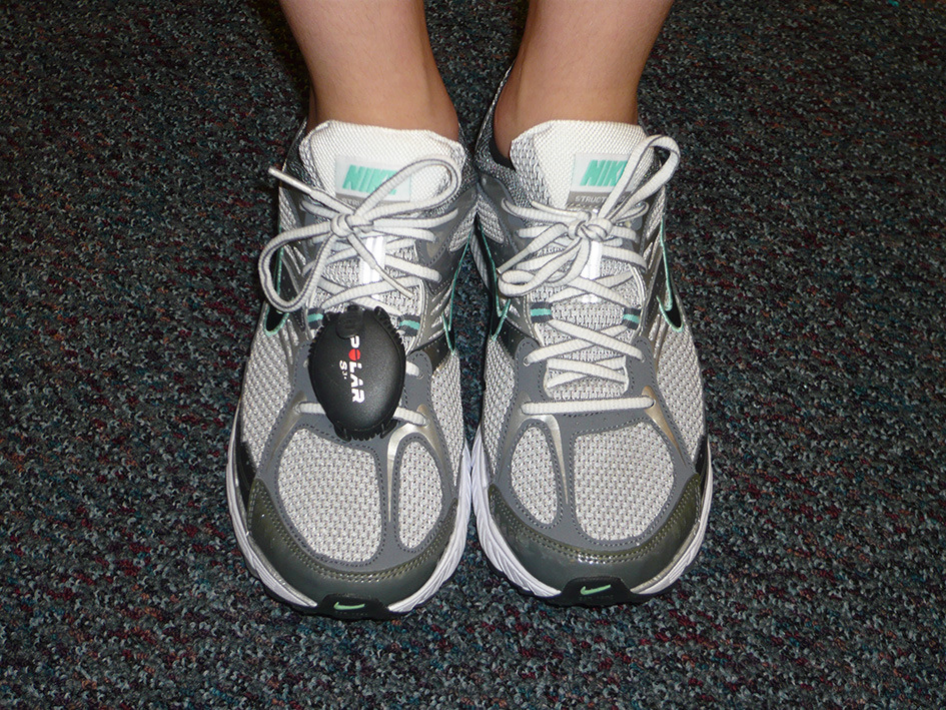
Placement of the S3+ Stride Sensor secured to the shoelaces of the right shoe.

On the first day of the season, participants warmed up by running ~1,600 m overground. Average running step rates were determined using Polar RCX5 wristwatches and S3+ Stride Sensors™ (Polar Electro, Inc., Lake Success, NY, USA) while runners ran individually, to avoid other runners influencing their speed or step rate, at their self-selected speed for 200 m on an outdoor track. The S3+ Stride Sensor was secured to the shoelaces of each runner's right shoe (Figure 1). Runners were cued to run the 200 m at the speed used for most of their weekly training mileage. The S3+ Stride Sensors™ have previously been shown to accurately and reliably determine step rate with a 1.4% error rate (2–3 steps per minute [spm]) (Hausswirth et al., 2009). The Polar S3+ Stride Sensor provided the mean strides per minute during the testing period and was set to sample at 1 Hz. The mean stride rate was doubled to determine steps per minute. Participants also completed a survey on demographics including age, height, weight, running experience, prior RRI, and summer running volume. A cohort of 68 high school cross country runners (47 females and 21 males; age = 16.2 ± 1.3 years, body mass 59.6 ± 9.0 kg, height = 168.1 ± 8.7 cm) was used for comparison of step rates at their self-selected speeds (Luedke et al., 2016).

### Injury Surveillance

Runners were followed during the intercollegiate season with RRI records for the runners kept by the university's athletic training staff using Athletic Trainer System® software. The date of RRI, body location of RRI, specific type of RRI, and days of missed or limited practice and/or competition were recorded. RRI data for participants was provided to the primary investigator by the head athletic trainer after the season ended. For this study, the occurrence of time-loss lower extremity RRI was used as the outcome measure. This was defined as a lower extremity medical problem resulting from running participation that required a runner to be removed from a practice or competitive event or to miss one or more subsequent practices or competitive events. Runners who were able to return to full, unrestricted participation before the end of the practice or meet were not considered injured in this study.

### Statistical Analysis

For collegiate runners, bivariate correlations were used to examine relationships between step rate and years of running experience, height, and body mass. Two sample *t*-tests were used to compare mean step rates in collegiate runners who did and did not experience a time-loss lower extremity RRI during the season. Initially, two sample t-tests were used to compare mean step rates between collegiate and high school runners; effect sizes were calculated using Cohen's *d*. Then analyses of covariance was used to compare mean step rates between collegiate and high school runners with self-selected speed, body mass, and both speed and body mass as co-variates. Chi-square analysis was used to compare proportions of collegiate and high school runners in low (≤166 spm), medium (167–177 spm), and high (≥178 spm) step rate categories as these were tertiles for high school runners at self-selected speeds (Luedke et al., 2016). All statistical analyses were performed using SPSS software (SPSS; v25.0, SPSS Inc., New York, USA).

## Results

In the 4 weeks prior to the first day of practice, our sample of collegiate runners reported running 6.2 ± 0.8 (range 4–7) days/week and 43.1 ± 15.2 (range 20–70) miles/week. Almost 90% (89.7%) reported running “pretty much the same” mileage each day for at least half of their runs and almost 75% of runners performed most of their pre-season training was on dirt, gravel or grass, while 27.6% reported half or more of their running was on concrete.

In our sample of collegiate runners, males were taller, heavier, and ran at faster self-selected speeds than the females (Table 1). Collegiate runners were older and taller than high school runners (Table 2). On average, collegiate runners had more years of running experience (Cohen's *d* = 1.34), ran at higher step rates (Cohen's *d* = 0.73), and at faster self-selected speeds (Cohen's *d* = 1.6) than high school runners (Table 2). While collegiate runners' self-selected mean step rates (177.1 ± 7.2 spm) were higher than high school runners' (171.3 ± 8.3 spm) (*p* = 0.01), the difference was non-significant when adjusted for higher self-selected speeds (collegiate [4.6 ± 0.5 m/s], high school [3.8 ± 0.5 m/s]; *p* = 0.19) (Table 2). When step rates were adjusted for body mass, the difference in step rates between high school and collegiate runners was significant (*p* < 0.001) (Table 2). The difference in step rates between high school and collegiate cross country runners was not statistically significant when running speed and body mass were simultaneously included as co-variates (*p* = 0.16) (Table 2).

**Table 1:** Baseline values of collegiate cross country runners.

**Variables**	**Total (*n* = 29)**	**Females (*n* = 13)**	**Males (*n* = 16)**	*p*-Value^*^
	**Mean ± SD**	**Mean ± SD**	**Mean ± SD**	
Age (years)	19.7 ± 1.3	19.7 ± 1.2	19.73 ± 1.3	0.87
Mass (kg)	60.9 ± 8.8	54.4 ± 6.0	66.1 ± 7.2	<0.001
Height (cm)	172.4 ± 8.5	165.4 ± 5.0	178.7 ± 5.6	<0.001
BMI (kg/m^2^)	20.4 ± 1.8	20.0 ± 1.8	20.7 ± 1.9	0.32
Running experience (years)	6.3 ± 1.5	7.0 ± 1.4	5.8 ± 1.5	0.02
Step rate at self-selected speed (spm)	177.1 ± 7.2	177.7 ± 8.2	176.6 ± 6.6	0.70
Self-selected speed (m/s)	4.6 ± 0.5	4.2 ± 0.4	4.9 ± 0.3	<0.001

*BMI, body mass index; spm, steps per minute*.

* *Two sample t-test of differences of mean values for females and males*.

**Table 2:** Comparison of baseline factors between high school and collegiate cross country runners.

	**High school runners (*n* = 68)**	**High school females (*n* = 47)**	**High school males (*n* = 21)**	**Collegiate runners (*n* = 29)**	**Collegiate females (*n* = 13)**	**Collegiate males (*n* = 16)**	*p*-value^*^	**Effect size (Cohen's *d*)^‡^**
	**Mean ± SD**	**Mean ± SD**	**Mean ± SD**	**Mean ± SD**	**Mean ± SD**	**Mean ± SD**		
Age (years)	16.2 ± 1.3^§^	16.2 ± 1.3	16.3 ± 1.5	19.7 ± 1.3^§^	19.7 ± 1.2	19.73 ± 1.3	<0.001	2.65
Mass (kg)	59.6 ± 9.0	57.1 ± 7.5	65.0 ± 9.8	60.9 ± 8.8	54.4 ± 6.0	66.1 ± 7.2	0.33	0.15
Height (cm)	168.1 ± 8.7^§^	164.3 ± 6.3	176.5 ± 7.1	172.4 ± 8.5^§^	165.4 ± 5.0	178.7 ± 5.6	0.02	0.50
BMI (kg/m^2^)	21.0 ± 2.7	21.1 ± 2.1	20.9 ± 3.7	20.4 ± 1.8	20.0 ± 1.8	20.7 ± 1.9	0.36	0.24
Running experience (years)	3.5 ± 2.3^§^	3.6 ± 2.4	3.1 ± 2.0	6.3 ± 1.5^§^	7.0 ± 1.4	5.8 ± 1.5	<0.001	1.34
Step rate at self-selected speed (spm)	171.3 ± 8.3^§^	172.6 ± 8.0	168.2 ± 8.3	177.1 ± 7.2^§^	177.7 ± 8.2	176.6 ± 6.6	0.001	0.73
Self-selected speed (m/s)	3.8 ± 0.5^§^	3.6 ± 0.5	4.1 ± 0.3	4.6 ± 0.5^§^	4.2 ± 0.4	4.9 ± 0.3	<0.001	1.6
Step rate adjusted for speed (spm)	172.1 ^å^	173.5^å^	170.6^å^	175.0^å^	174.7^å^	173.5^å^	0.19^†^	
Step rate adjusted for body mass (spm)	171.1^å^	172.8^å^	168.1^å^	177.4^å^	177.0^å^	176.8^å^	<0.001^†^	
Step rate adjusted for speed and body mass (spm)	172.1^å^	173.5^å^	169.9^å^	175.0^å^	174.5^å^	174.4^å^	0.16^†^	

*BMI, Body mass index; spm, steps per minute*.

* *Two sample t-test of differences of mean values for high school and collegiate runners*.

‡ *Effect sizes for comparisons between high school and collegiate runners*.

§ *Significant difference between total samples of high school and collegiate runners*.

† *ANCOVA with step rate between school and collegiate runners adjusted for speed and/or body mass*.

å *Adjusted means from ANCOVA analysis*.

*High school sample from Luedke et al. (2016)*.

Using step rate classifications from an analysis of high school runners at self-selected speeds (≤166, 167–177, ≥178 steps/min) (Luedke et al., 2016), there was a significantly lower percentage (6.9%) of collegiate runners who ran at ≤166 spm than high school runners (30.9%) (*p* = 0.01) (Table 3). The percentage of collegiate runners with step rates ≥178 spm (48.3%) was not significantly higher than for high school runners (30.9%) (*p* = 0.10) (Table 3).

**Table 3:** Proportions of runners with step rates ≤166 and ≥178 spm between collegiate and high school runners.

	**High school cross country runners (*n* = 68)**	**Collegiate cross country runners (*n* = 29)**
	***n* (%)**	***n* (%)**
**Step rate at self-selected speed**	**Mean self-selected speed = 3.8 ± 0.5 m/s**	**Mean self-selected speed = 4.6 ± 0.5 m/s**
≤166 spm	21 (30.9)^*^	2 (6.9)^*^
167–177 spm	26 (38.2)	13 (44.8)
≥178 spm	21 (30.9)	14 (48.3)

*spm, steps per minute*.

* *p < 0.05 Chi-square analysis to compare proportions*.

*High school sample from Luedke et al. (2016)*.

Among the collegiate runners, body mass was negatively correlated with step rate (*r* [95% CI] = −0.43 [−0.69, −0.08]; *p* = 0.02) (Table 4). Years of running experience (*r* = 0.04 [−0.32, 0.41]; *p* = 0.81) and height (*r* = −0.30 [−0.60, 0.08]; *p* = 0.11) were not significantly correlated to self-selected step rate.

**Table 4:** Relationships between collegiate runner running experience, selected anthropometric variables, and step rate at self-selected speed [Pearson *r* correlation values (95% CI)].

**Variables**	**Total (*n* = 29)**	**Females (*n* = 13)**	**Males (*n* = 16)**
	**Step rate at self-selected speed**	**Step rate at self-selected speed**	**Step rate at self-selected speed**
Experience	0.05 (−0.32, 0.41)	0.21 (−0.39, 0.68)	−0.16 (−0.61, 0.37)
Mass	−0.43^*^ (−0.69, −0.08)	−0.43 (−0.79, 0.16)	−0.60^*^ (−0.85, −0.15)
Height	−0.30 (−0.60, 0.08)	−0.43 (−0.79, 0.16)	−0.40 (−0.75, 0.12)
BMI	−0.36 (−0.64, 0.01)	−0.24 (−0.70, 0.36)	−0.48 (−0.79, 0.02)
Self-selected speed	0.12 (−0.26, 0.47)	0.18 (−0.41, 0.67)	0.38 (−0.14, 0.74)

*BMI, body mass index*.

* *p < 0.05*.

With respect to prior injury, the mean step rate (175.5 ± 7.3 spm) of collegiate runners who reported a lower extremity RRI in the prior year was similar to the mean step rate of collegiate runners who did not have a lower extremity RRI in the prior year (178.9 ± 6.9 spm) (*p* = 0.21). During the season, 12 (41.4%) of the collegiate runners experienced a lower extremity RRI. The mean step rate for collegiate runners who did not experience a lower extremity RRI during the cross country season (178.9 ± 7.7 spm) was not significantly higher than the mean step rate for collegiate runners who did experience a lower extremity RRI (174.5 ± 5.7 spm) (*p* = 0.10). Of the 12 time-loss lower extremity RRIs, the most common body locations affected were the lower leg (58.3%) and ankle/foot (16.7%). The most common specific injury types were calf strain/Achilles tendinopathy (33.3%) and medial tibial stress syndrome (25.0%) There were also individual injury type cases of anterior knee pain, gluteal tendinopathy, iliotibial band compression syndrome, ankle sprain, and metatarsal stress syndrome.

## Discussion

The purpose of our study was to compare step rates between collegiate and high school cross country runners. Consistent with our hypothesis, on average, collegiate runners ran at a higher self-selected step rate than high school runners. The difference in step rates between collegiate and high school runners was significant when step rate was adjusted for body mass. However, when adjusting for self-selected speeds, the mean step rates were similar and no longer statistically significant. This suggests that the higher step rate in collegiate runners was at least partially explained by higher self-selected running speeds compared to the high school runners.

As expected, collegiate runners had significantly more years of running experience than high school runners. Additional years of running experience may lead collegiate runners to be more proficient runners. Alternatively, as only 5.3% of male and 7.1% of female high school cross country runners go on to participate in NCAA collegiate cross country (National Colllegiate Athletic Association, 2020), collegiate samples may include more proficient runners. The high school sample used for comparison included less experienced runners; novice runners tend to self-select step rates on average 8% lower than their metabolically optimal step rates while trained runners with 10k personal bests of 34 min 53 s ± 85 s ran at step rates closer to their optimal step rate than novice runners (de Ruiter et al., 2014). At matched speeds from 10 to 17 km/h, trained runners ran with significantly higher step rates than untrained participants (Gomez-Molina et al., 2017). Trained adult runners (>20 miles/week for >4 weeks) self-selected step rates that approximated the most optimal metabolically efficient step rates (Hunter and Smith, 2007). With increased experience, highly trained runners generally run with higher step rates and less vertical excursion of center of mass (Slawinski and Billat, 2004). Thus, those who elect to compete in collegiate cross country may be more likely to have developed gait parameters that balance both metabolic demand and impact forces.

A difference in step rates between high school and collegiate runners might be partially due to coaching recommendations at the different levels. While not formally assessed, in discussions with the head coaches of the high school and collegiate teams, the coaches shared that cadence was not something they specifically included in their cueing or instructions on form to their athletes. Coaches or other sources of information that runners access referencing cadence may influence step rate but the literature on that relationship is limited.

Another purpose of this study was to determine if step rates in collegiate runners were related to experience and anthropometric variables. Body mass was negatively correlated to step rate in the total sample of collegiate runners and in male collegiate runners. Higher step rates necessitate increased hip flexor moments, or power, to accelerate the leg (Lieberman et al., 2015) and demands may further increase in those with greater mass. Based on these findings, coaches may expect to see variations in step rate based on body mass in collegiate runners. Running experience and height were not significantly correlated to step rate in collegiate runners. Our finding that years of running experience was not significantly related to step rate in collegiate runners is in contrast to reports in adult (Slawinski and Billat, 2004; Gomez-Molina et al., 2017) and adolescent cross country runners (Luedke et al., 2018). The lack of relationship in our collegiate sample may have been due to the homogeneity of running experience values in the group and the variation in step rate values. We expected greater height to be linked with longer step lengths and thus lower step rates at a set speed based on prior research (Elliott and Blanksby, 1979; Cavanagh and Kram, 1989; Luedke et al., 2018). Limited variation in collegiate runners' heights may have also accounted for this non-relationship.

Lastly, we aimed to determine whether the self-selected step rates of collegiate runners were related to lower extremity RRI during the season. The mean step rate for those who experienced a lower extremity RRI during the season was not significantly lower than the mean step rate of those who did not experience a lower extremity RRI. There may be several possible reasons for the non-significant relationship. First, the small sample size of runners likely did not allow adequate power to detect a significant association. Second, we observed that a significantly smaller proportion of collegiate runners displayed step rates that may be considered low (≤166 spm) than high school runners and this may partially explain the non-significant relationship between low step rate and RRI in the collegiate sample. Third, our results differ from results in high school runners whereby all RRI were included in the analysis rather than just anterior knee pain and shin injuries (Luedke et al., 2016). The non-significant relationship between step rate and RRI in the present study is consistent with findings of Szymanek et al. who also assessed the relationship between step rate and any RRIs and did not find a significant relationship (Szymanek et al., 2020). Perhaps, lower step rates may increase susceptibility of specific body locations or injury types as step rate affects joint loading and impact forces (Schubert et al., 2013). While shin injury was associated with step rate in high school runners (Luedke et al., 2016), the present sample only had an occurrence of three shin injuries. One-third of injuries were to the calf or Achilles tendon and Achilles stress and strain is more likely influenced by foot strike than step rate (Lyght et al., 2016).

Several strengths of the current study are noteworthy. This study used a prospective design where the risk factor variables (demographic, anthropometric, experience, step rate) were assessed on the first day of the season and prior to injury, thus minimizing any recall or measurement biases. Another strength is that our study assessed step rate overground instead of on a treadmill as cross country training and competition is typically conducted overground. While a recent meta-analysis suggested no significant differences between step rates during overground and treadmill running, the studies included had inconsistent findings and were rated very low to low quality (Van Hooren et al., 2020). Wearable sensors like the Polar S3+ Stride Sensor used in this study appear suitable to assess step rate during overground running with step rate as a potential factor in RRI. Male recreational runners improved kinetic variables linked with injury over 12 weeks of step rate manipulation (Wang et al., 2020). In addition to assessing self-selected step rates as in the present study, Stride Sensors can be used to provide real time feedback on step rate while running. Beyond real time step rate assessment, another potential use for step rate tracking is with respect to quantifying external training load. Multiplying the average step rate by the duration of a run may provide a more precise measure of external training load than just distance run which is commonly used (Paquette et al., 2020).

Several limitations of this study are also noted. First, our study sample was comprised of Division III collegiate runners from one university so findings may not be generalizable to runners in other collegiate settings. Thus, future studies of male and female cross country runners from all collegiate divisions are recommended for comparative purposes. Second, a potential limitation is that speed was not controlled in the step rate assessment. Assessing step rate at set speeds controls for the effect of speed; however, we opted to use self-selected speed to ascertain the step rate likely used during most of weekly training mileage during the season. Third, while the runners followed similar training schedules, the primary exposure of running volume was not taken into account for injuries. Fourth, when step rate was assessed on the first day of the season, it did not account for any changes that may have occurred in step rate during the season. Finally, while we ascertained data on RRIs in the collegiate runners, our sample size did not allow adequate power to examine the risk relationship between step rate and RRI as had been assessed in a prior study of high school cross-country runners (Luedke et al., 2016). As most RRIs in this study were overuse in nature as in their study, a larger sample of collegiate runners' step rates is recommended to appropriately evaluate step rate as a risk factor for RRI in collegiate runners, as well as for further comparisons to high school cross country runners.

In conclusion, higher step rates were observed in collegiate compared to high school cross country runners partially due to increased running speeds. As body mass was significantly correlated to step rate, step rate recommendations should consider multiple factors that influence step rate.

## Data Availability Statement

The raw data supporting the conclusions of this article will be made available by the authors, without undue reservation.

## Ethics Statement

The studies involving human participants were reviewed and approved by University of Wisconsin Oshkosh Institutional Review Board. The patients/participants provided their written informed consent to participate in this study.

## Author Contributions

LL and MR: conceptualization, methodology, data curation, and writing—review and editing. LL: formal analysis, investigation, and writing—original draft preparation. Both authors have read and agreed to the published version of the manuscript.

## Conflict of Interest

The authors declare that the research was conducted in the absence of any commercial or financial relationships that could be construed as a potential conflict of interest.
